# Chemerin Stimulates the Secretory Activity of BME-UV1 Bovine Mammary Epithelial Cells

**DOI:** 10.3390/ijms25084147

**Published:** 2024-04-09

**Authors:** Żaneta Dzięgelewska-Sokołowska, Alicja Majewska, Iwona Szopa, Małgorzata Gajewska

**Affiliations:** Department of Physiological Sciences, Institute of Veterinary Medicine, Warsaw University of Life Sciences (SGGW), Nowoursynowska 159b, 02-776 Warsaw, Poland; zaneta_dziegelewska-sokolowska@sggw.edu.pl (Ż.D.-S.); alicja_majewska@sggw.edu.pl (A.M.); iwona_szopa@sggw.edu.pl (I.S.)

**Keywords:** bovine mammary epithelial cells, chemerin, leptin, adiponectin, viability, apoptosis, αS1-casein

## Abstract

Adipose tissue is an active endocrine gland, synthesizing and secreting multiple signaling molecules termed adipokines. Following the detection of adipokines and their receptors in the mammary tissue of various species, it is indicated that adipokines play a role in the development of the mammary gland. The aim of the present study was to determine the concentration-dependent influence of three adipokines, leptin, adiponectin, and chemerin, on the viability, apoptosis, and secretory activity of BME-UV1 bovine mammary epithelial cells. The study confirmed that BME-UV1 cells contain the leptin receptor (Ob-R) protein, and express transcripts of adiponectin (*ADIPOR1* and *ADIPOR2*) and chemerin (*CMLKR1* and *GPR1*) receptors. Regardless of the administered dose, none of the three tested adipokines had an effect on the viability of BME-UV1 cells, and the number of apoptotic cells remained unchanged. However, chemerin (100 ng/mL) stimulated BME-UV1 cells to synthesize and secrete αS1-casein, the major protein component of milk. These results indicate that chemerin may be a potent regulator of the bovine mammary epithelial cells’ functional differentiation, contributing, along with the major systemic hormones and local growth factors, to the development of the bovine mammary gland.

## 1. Introduction

The mammary gland is a highly evolved and specialized exocrine gland characteristic of mammals. The parenchyma of the mammary gland is composed of mammary epithelial cells (MECs) that synthesize and secrete milk during lactation, when the gland shows full functional development. The MEC bilayer comprises luminal cells, localized internally, and myoepithelial (basal) cells, localized externally [1,2]. This branching network of ducts and lobuloalveolar structures is encased by a basement membrane and embedded in the stroma [3]. The stromal compartment of the mammary gland plays the role of a scaffold for the parenchymal tissue, supplies nutrients and substrates for the synthesis of milk components, and provides cells and cytokines important for immune defense [4].

The heterogenous mammary gland stroma is composed of white adipocytes, fibroblasts, but also a variety of immune cells, endothelium, and a neural network, that all form a milieu regulating the progress of the mammary epithelium development [5]. Among all cells of the mammary gland, stromal adipocytes create the most abundant niche. Currently, adipose tissue is believed to represent one of the major endocrine glands, and is a rich source of biologically active compounds, termed adipokines, acting in a paracrine, juxtacrine, and endocrine manner [6].

The expression of many adipokines has been confirmed in the mammary gland stromal adipose tissue. Among them, leptin and adiponectin are the most studied ones and exert opposite effects. Leptin is a 16 kDa adipokine encoded by the obesity (Ob) gene and produced primarily by white adipocytes [7]. This hormone regulates body energy balance, suppresses food intake, and also influences reproductive functions [8]. Leptin acts on target cells through transmembrane receptor (Ob-R), which exists in six different isoforms (from Ob-Ra to Ob-Rf), expressed mainly in the hypothalamic neurons, gonadotrope cells of the anterior pituitary, and interstitial cells of the ovary and endometrium [8,9]. The expression of leptin receptors has been detected in the MEC of various species, suggesting that the proliferation and differentiation of epithelial cells may be also controlled by leptin [10,11]. Deficiency in leptin signaling caused by a mutation in the leptin gene resulted in the severe underdevelopment of the mammary tissue [12,13]. In addition, studies have shown that the synthesis of leptin by mammary adipose tissue is regulated by systemic hormones, such as insulin, sex steroids, glucocorticoids, and prolactin [14].

Adiponectin is a 30 kDa adipokine encoded by the *AdipoQ* gene. It is synthesized and secreted mainly by white adipose tissue, and found at high concentrations in blood plasma [15]. Adiponectin increases the insulin sensitivity of target cells, regulating glucose intake as well as the differentiation of various cell types. Adiponectin also shows anti-inflammatory and antioxidant activities [15]. The expression of adiponectin has been confirmed in the parenchymal and stromal compartment of the bovine mammary gland during lactation, suggesting the role of this adipokine in the paracrine regulation of MEC differentiation [16]. Adiponectin acts via two different transmembrane receptors, AdipoR1 and AdipoR2. The expression of both receptors was detected in normal mammary epithelial cells [9,14]. Leptin and adiponectin’s opposing action is well described in the mammary gland tissue. Esper et al. [17] showed that leptin and adiponectin may be involved in the regulation of the size of mammary stem cell (MaSC) population within the glandular tissue, which is crucial for cell renewal. The number of MaSCs was directly proportional to the leptin/adiponectin ratio [17].

Studies conducted in recent years revealed that chemerin may be one of the essential adipokines regulating the development and function of the mammary epithelium [18,19]. Chemerin, also known as retinoic acid receptor responder protein 2 (RARRES2), or tazarotene-induced gene 2 protein (TIG2), is a 16 kDa chemoattractant cytokine (chemokine) secreted mainly by white adipose tissue [20]. Three receptors are able to bind chemerin with high affinity, namely, GPR1 (G protein-coupled receptor 1), CMKLR1 (chemokine receptor-like 1 or ChemR23), and CCRL2 (C-C chemokine receptor-like 2 or CC motif) [20,21]. Chemerin and its receptors are detected in different tissues (i.e., spleen, fetal liver, lymph nodes, and bone marrow), indicating its multifunctional role [21]. Recent studies have shown the expression of chemerin and its two receptors, CMKLR1 and CCRL2, in MAC-T bovine mammary epithelial cells and in the mammary gland tissue samples derived from Holstein dairy cows [18]. The treatment of MAC-T cells with chemerin caused an increased expression of genes involved in milk protein synthesis, glucose uptake, and fatty acid synthesis, suggesting that chemerin may regulate the process of milk synthesis in the bovine mammary gland [18]. In another study, Suzuki et al. [19] demonstrated that chemerin supports bovine MEC growth and epithelial barrier function and is strongly regulated by inflammatory stimuli, such as TNFα.

Information about the role of bioactive molecules produced by adipocytes in the regulation of the bovine mammary epithelium development is still insufficient, despite the prime contribution of this species in global milk production [22]. Our previous in vitro study on primary bovine mammary epithelial cells (bMECs) and primary bovine adipocytes showed that conditioned media (CM) derived from mature bovine adipocytes stimulated formation of mammospheres by bMECs cultured on Matrigel, and significantly decreased the number of apoptotic cells in the bMEC population [23]. An immunoenzymatic analysis of the composition of collected CM confirmed the presence of adiponectin, leptin, and chemerin in the media [22]. Therefore, in the present study, we aimed to investigate the effect of leptin, adiponectin, and chemerin on the viability and functions of bovine BME-UV1 mammary luminal epithelial cells [24]. In this study, BME-UV1 cells were exposed to low and high concentrations of adipokines to investigate their effect on cell viability, apoptosis, and functional differentiation, assessed by the ability of bovine MECs to synthesize and secrete milk proteins.

## 2. Results

### 2.1. Effect of Chemerin, Leptin, and Adiponectin on the Viability of BME-UV1 Cells

**Figure 1:**
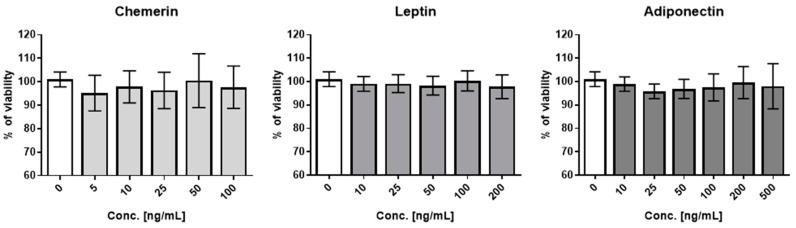
Viability of BME-UV1 bovine mammary epithelial cells treated for 24 h with different concentrations of chemerin, leptin, or adiponectin. Graphs present cell viability measured using the MTT assay. The viability of untreated control cells (0) was designated as 100%. Results are presented as means ± standard deviation of three independent experiments.

Our recent studies have shown that primary bovine adipocytes, differentiating from bovine adipocyte-derived stem cells (bASCs), synthesize and secrete chemerin, leptin, and adiponectin in an in vitro culture. The conditioned media derived from bASCs regulated bovine MEC viability and secretory activity [23]. In the present study, we investigated the effect of chemerin, leptin, and adiponectin on vital functions of BME-UV1 cells, representing the luminal lineage of bovine mammary epithelial cells. In the first step of our research, we used the MTT assay to choose the optimal concentrations of each tested adipokine, that did not cause a cytotoxic effect in the bovine MECs. The viability of untreated control cells was denoted as 100%. The results of the MTT assay did not show any significant differences in the viability of cells treated with a range of concentrations of chemerin (5, 10, 25, 50, and 100 ng/mL), leptin (10, 25, 50, 100, and 200 ng/mL), or adiponectin (10, 25, 50, 100, 200, and 500 ng/mL) (Figure 1).

In the subsequent part of our research, we selected two concentrations (low and high) of each adipokine: chemerin (10, 100 ng/mL), leptin (10, 100 ng/mL), and adiponectin (10, 500 ng/mL) based on available data of other studies using in vitro models [17,18,24,25,26,27]. The high concentration of adiponectin (500 ng/mL) differed from the chosen values of high doses of the two other adipokines (100 ng/mL), because adiponectin levels in circulating blood are expressed in µg/mL, whereas in bovine milk, adiponectin can be detected at a concentration of 600 ± 30 ng/mL [26]. Therefore, we chose a high dose of adiponectin (500 ng/mL) that resembled the physiological concentration detected in bovine milk.

### 2.2. Expression of Chemerin, Leptin, and Adiponectin Receptors in BME-UV1 Cells

**Figure 2:**
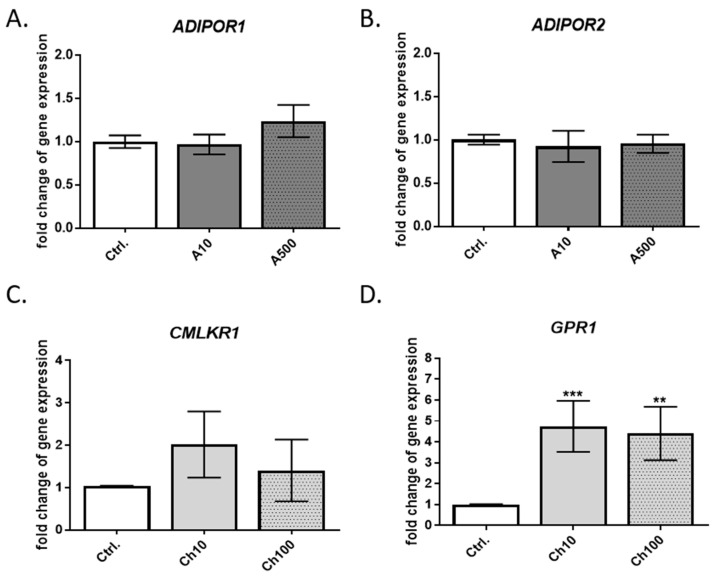
Expression of adipokine receptors in BME-UV1 bovine mammary epithelial cells. (**A**,**B**) Expression of adiponectin receptors (*ADIPOR1* and *ADIPOR2*); (**C**,**D**) expression of chemerin receptors (*CMLKR1* and *GPR1*). The relative mRNA expression of the analyzed genes was normalized to the mean expression of the histone reference gene. The expression of each analyzed gene in cells cultured in control medium (Ctrl.) was given as 1. Treatment with adiponectin at concentrations of 10 ng/mL or 500 ng/mL is marked as A10 and A500, respectively. Treatment with chemerin at concentrations of 10 ng/mL or 100 ng/mL is marked as Ch10 and Ch100, respectively. Results are presented as means ± standard deviation of three independent experiments performed in duplicate. Values that differed significantly from control are marked as ** (*p* < 0.01) or *** (*p* < 0.001).

To confirm the expression of leptin, adiponectin, and chemerin receptors in BME-UV1 cells, we used reverse transcription-quantitative PCR (RT-qPCR). The expression of genes encoding: adiponectin receptors *ADIPOR1* and *ADIPOR2,* chemerin receptors *CMLKR1, CCRL2,* and *GPR1*, and leptin receptor *OB-R* was analyzed in untreated control cells and in bovine MECs treated for 24 h with low and high doses of adiponectin (10, 500 ng/mL), chemerin (10, 100 ng/mL), or leptin (10, 100 ng/mL). Transcripts of both adiponectin receptors, *ADIPOR1* and *ADIPOR2*, were detected in BME-UV1 cells. The addition of adiponectin did not significantly affect the expression of either gene, regardless of the administered dose (Figure 2A,B).

Among the three analyzed transcripts of chemerin receptors, only two were detected in BME-UV1 cells, namely, *CMLKR1* and *GPR1*. The expression of *GPR1* was over two times higher than *CMLKR1,* and it increased significantly in the presence of chemerin added to the culture medium at low and high concentrations (*p* = 0.0008 and *p* = 0.0023, respectively) (Figure 2C,D).

**Figure 3:**
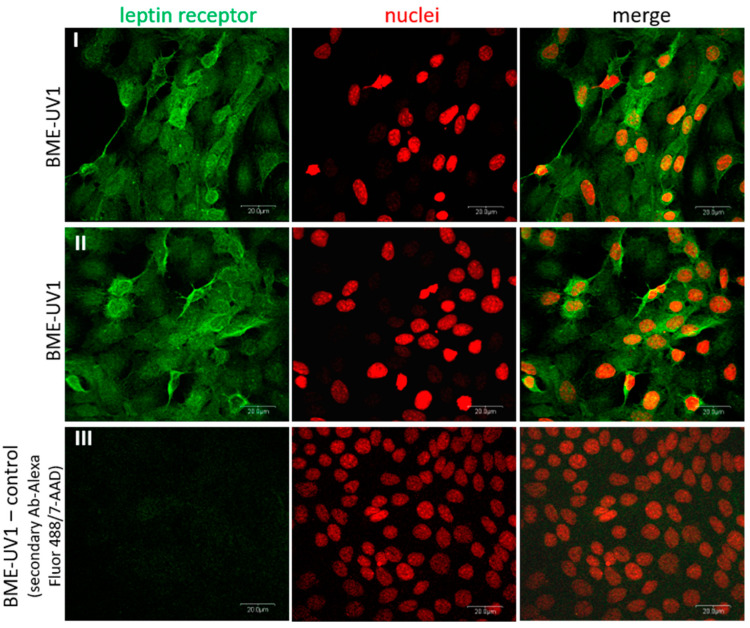
Immunofluorescence staining of leptin receptor in BME-UV1 bovine mammary epithelial cells. Panels I and II present the localization of leptin receptor detected using primary antibodies (cat. no. bs-0961; Bioss Antibodies), followed by secondary antibodies conjugated with Alexa Fluor 488 dye (green fluorescence); nuclei were counterstained with 7-amino actinomycin (7-AAD, red fluorescence). Panel III presents no primary antibody control. Micrographs were taken at 600× magnification. Scale bar: 20 μm.

Unexpectedly, the RT-qPCR analysis did not confirm the expression of gene encoding leptin receptor (*OB-R*) in BME-UV1 cells. We tested four different primer pairs (one commercial and three pairs published by other authors) at different annealing temperatures and concentrations (Table 1), with no success in amplification. Thus, the expression of bovine leptin receptor was additionally analyzed using immunofluorescence staining. BME-UV1 cells showed positive staining for leptin receptor. A high intensity of green fluorescence was observed at the outer plasma membrane region, confirming the cell membrane localization of OB-R (Figure 3). The specificity of staining was confirmed by a control staining in which the cells were labelled only with the secondary antibodies conjugated with Alexa Fluor 488 dye. No labeling was detected in the control staining (Figure 3).

### 2.3. Effect of Chemerin, Leptin, and Adiponectin on Apoptosis Induction in BME-UV1 Cells

**Figure 4:**
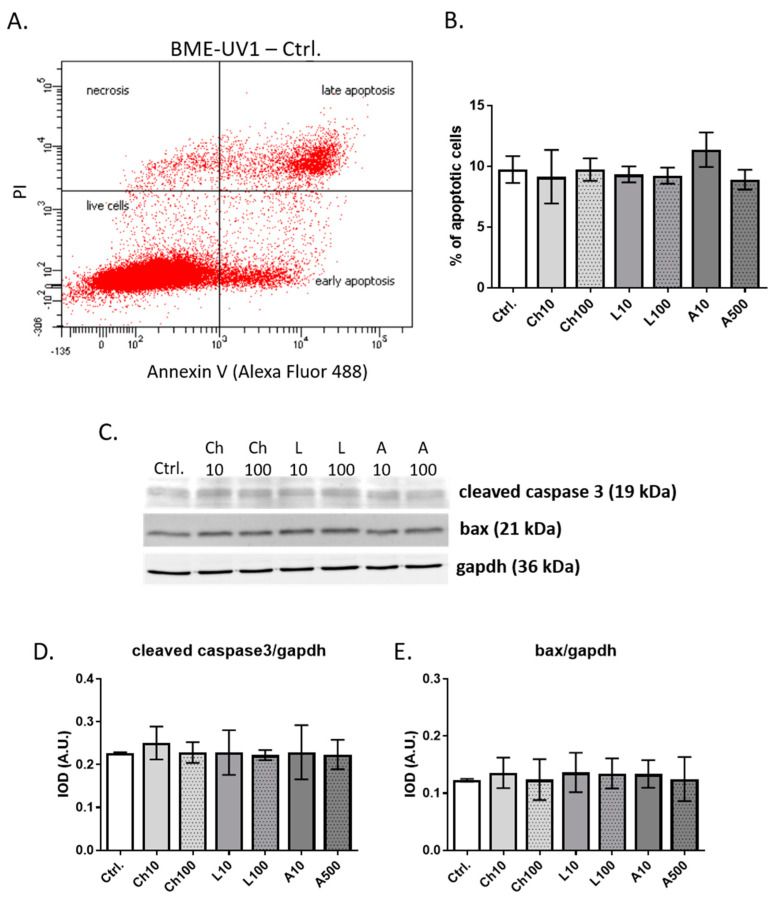
Evaluation of apoptosis in BME-UV1 bovine mammary epithelial cells treated with chemerin (Ch10 = 10 ng/mL, Ch100 = 100 ng/mL), leptin (L10 = 10 ng/mL, L100 = 100 ng/mL), or adiponectin (A10 = 10 ng/mL, A500 = 500 ng/mL) for 24 h. (**A**) Representative dot-plots of Annexin V/PI double staining in untreated control cells; (**B**) graph showing percentage of apoptotic cells (sum of Annexin V^pos^/PI^neg^ and Annexin V^pos^/Pi^pos^ cells) in control and adipokine-treated cells; (**C**) representative images of Western blot (WB) analysis of apoptotic markers cleaved caspase 3 and bax, where gapdh was used as a reference protein; (**D**,**E**) graphs showing the results of the densitometric analysis of WB images; the integrated optical density (IOD) of cleaved caspase 3 and bax bands was normalized to the IOD of gapdh bands. Results are presented as means ± standard deviation of three or four independent experiments.

After confirming the expression of receptors specific for the investigated adipokines, in the next step of our research we analyzed the effect of chemerin, leptin, and adiponectin on apoptosis induction in BME-UV1 cells. The percentage of apoptotic cells in the bovine MEC population was determined using the Annexin V assay and flow cytometry (Figure 4). No significant changes in the percentage of apoptotic cells (Annexin V^pos^/PI^neg^ and Annexin V^pos^/PI^pos^) were noted in the population of BME-UV1 cells treated with chemerin (10 ng/mL and 100 ng/mL), leptin (10 ng/mL and 100 ng/mL), or adiponectin (10 ng/mL and 500 ng/mL) in comparison to control conditions (untreated cells) (Figure 4B). Furthermore, Western blot analysis did not show any significant changes in the expression of two apoptotic markers, bax and cleaved caspase-3, confirming the results of the Annexin V assay (Figure 4C–E).

### 2.4. Effect of Chemerin, Leptin, and Adiponectin on the Secretory Activity of BME-UV1 Cells

**Figure 5:**
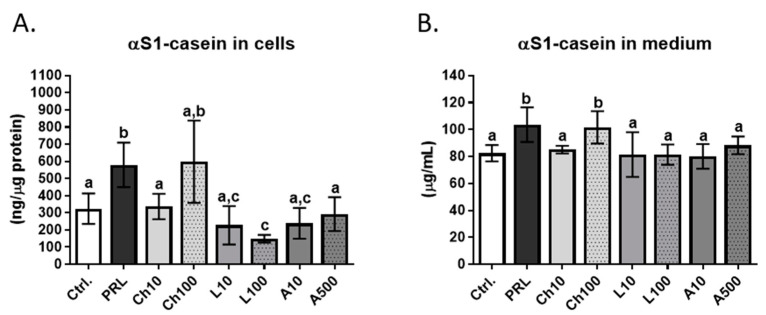
Concentration of αS1-casein in BME-UV1 cells (**A**) and media (**B**) collected after 24 h of culture. Cell were treated with chemerin (Ch10 = 10 ng/mL, Ch100 = 100 ng/mL), leptin (L10 = 10 ng/mL, L100 = 100 ng/mL) or adiponectin (A10 = 10 ng/mL, A500 = 500 ng/mL). Untreated cells were used as a negative control, whereas cells exposed for 24 h to the lactogenic hormone prolactin (PRL, 1 µg/mL) were used as a positive control. Results are presented as means ± standard deviation of three independent experiments. Means followed by a common letter are not significantly different according to one-way ANOVA with Tukey’s multiple comparison post-test, at the 5% level of significance (*p* < 0.05).

An essential feature of the functional differentiation of the mammary epithelial cells during lactation is their ability to synthesize and secrete milk proteins under the influence of lactogenic hormones. In the present study, αS1-casein, one of the major bovine milk proteins, was chosen as a marker of the functional differentiation of BME-UV1 cells [31,32]. The concentration of αS1-casein was determined using ELISA in cells treated for 24 h with chemerin (10 and 100 ng/mL), leptin (10 and 100 ng/mL), or adiponectin (10 and 500 ng/mL), and in the media collected after 24 h incubation with adipokines. The effect of adipokines on bovine MEC secretory activity was compared with the effect of the lactogenic hormone prolactin (PRL, 1 μg/mL) that served as a positive control [33], and with basic control conditions (untreated cells). The concentration of prolactin was chosen based on scientific reports published by other research groups [25,32,34]. As expected, the treatment of BME-UV1 cells with PRL caused a significant increase in the synthesis and secretion of αS1-casein (Figure 5). In the presence of adiponectin, the concentration of αS1-casein in cells and the conditioned media (CM) did not differ significantly from the level detected in the untreated cells, and was significantly lower than in PRL-treated cells. Treatment with leptin did not stimulate the secretion of αS1-casein above the levels detected in CM collected from the untreated control cells, but the concentration of this milk protein differed significantly from that detected in CM from cells incubated with PRL. The higher dose of leptin (100 ng/mL) caused a significant decline in the concentration of αS1-casein measured in cells in comparison to the basic control (untreated cells), as well as the positive control treated with PRL (Figure 5A). In the case of chemerin, the treatment of BME-UV1 cells with the low concentration (10 ng/mL) did not affect the synthesis and secretion of αS1-casein, which was at a similar level to the untreated control; however, at the higher dose (100 ng/mL), chemerin significantly stimulated the expression of this milk protein. The concentrations of αS1-casein in cells exposed to 100 ng/mL of chemerin and in the collected conditioned media were similar to the levels detected in bovine MECs treated with PRL (Figure 5). Information about the values of adjusted *p* value for comparisons that were statistically significant (*p* ≤ 0.05) in the Tukey’s multiple comparison post-test are presented in Supplementary Table S1.

### 2.5. The Role of Chemerin, Leptin. and Adiponectin in the Regulation of Mammosphere Formation by BME-UV1 Cells Cultured on Matrigel

**Figure 6:**
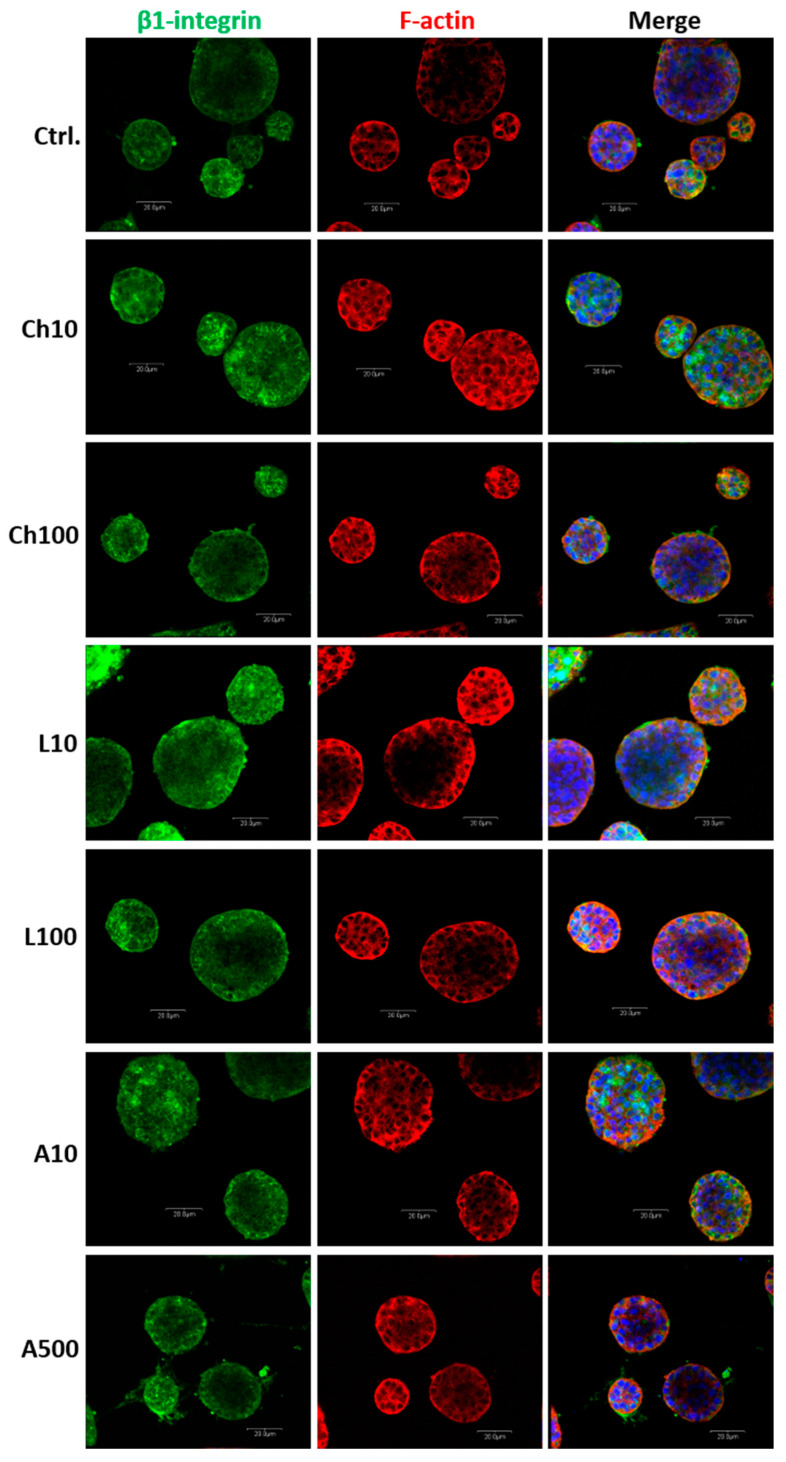
Panels of confocal micrographs presenting the immunofluorescence staining of BME-UV1 cells cultured on Matrigel for 11 days in control growth medium (Ctrl.) or medium supplemented with chemerin (Ch10 = 10 ng/mL, Ch100 = 100 ng/mL), leptin (L10 = 10 ng/mL, L100 = 100 ng/mL), or adiponectin (A10 = 10 ng/mL, A500 = 500 ng/mL). Cells were stained with primary antibodies against β1-integrin and secondary antibodies conjugated with Alexa Fluor 488 dye (green fluorescence); Alexa Fluor 594 phalloidin, detecting F-actin (red fluorescence), and nuclei counterstained with Hoechst 33342 (blue fluorescence). Images were taken at 600× magnification and are representative for three independent experiments. Scale bar: 20 μm.

**Figure 7:**
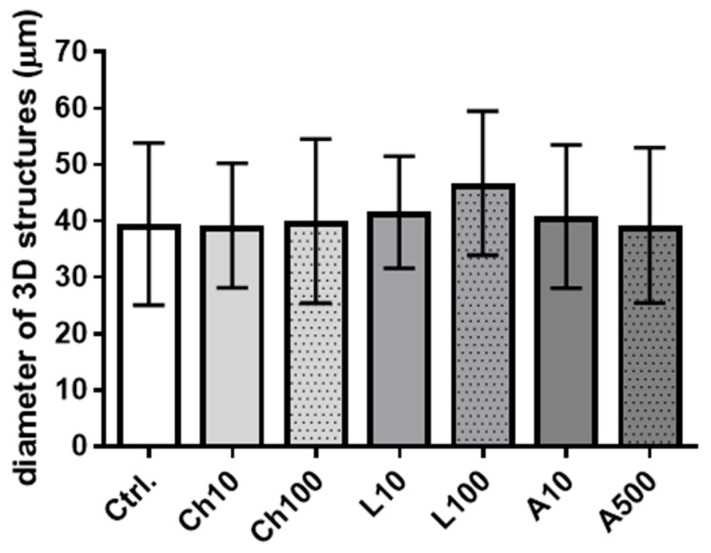
Diameters of 3D mammospheres formed by BME-UV1 cells cultured on Matrigel for 11 days. Cells were cultured in control growth medium (Ctrl.) or medium supplemented with chemerin (Ch10 = 10 ng/mL, Ch100 = 100 ng/mL), leptin (L10 = 10 ng/mL, L100 = 100 ng/mL), or adiponectin (A10 = 10 ng/mL, A500 = 500 ng/mL). The diameters of cross sections of the spheroids were measured using ImageJ software (https://ij.imjoy.io accessed on 2 April 2024). Results are presented as means ± standard deviation of at least 14 spheroids per experimental condition photographed using a confocal microscope equipped with a digital camera.

The results obtained encouraged us to determine whether the investigated adipokines are involved in the regulation of the alveologenesis process. During pregnancy, the mammary gland epithelium forms alveoli that represent the basic secretory units at the time of lactation. Thus, we analyzed the ability of BME-UV1 cells to form three dimensional (3D) spherical structures, called mammospheres, when cultured on reconstituted basement membrane (Matrigel), and treated with chemerin, leptin, or adiponectin. Confocal images of the 3D structures formed by BME-UV1 cells did not reveal any noticeable differences in the shape of mammospheres formed by untreated control cells and by bovine MECs exposed to the adipokines. The pattern of localization of β1-integrin and F-actin was also similar in all tested conditions (Figure 6). In addition, the size of mammospheres did not differ among the experimental and control conditions (Figure 7).

## 3. Discussion

Studies have shown that adipokines and their specific receptors are expressed in the mammary tissue, suggesting the role of these bioactive molecules in the mammary gland development [35]. Adipokines may exert their effect on the mammary parenchyma through endocrine mechanisms, being transported to the gland with circulating blood, but these bioactive molecules may also be synthesized locally by the adipose tissue of the mammary stroma, regulating the growth and metabolism of MECs in a paracrine manner [35,36].

In the first step of our research, BME-UV1 cells were exposed to a range of concentrations of leptin (10–200 ng/mL), adiponectin (10–500 ng/mL), or chemerin (5–100 ng/mL) to assess the viability of bovine MECs in the presence of the tested adipokines. The results of the MTT assay did not show a significant effect of the investigated adipokines on the BME-UV1 cells’ metabolic activity at any of the applied concentrations. Thus, the concentrations of leptin, adiponectin, and chemerin used in our further experiments were chosen based on the available literature. Studies on in vitro models of bovine MECs used leptin in a dose range of 10, 50, or 100 ng/mL [25,27], chemerin at a concentration range of 10, 100, or 300 ng/mL [18,19], and adiponectin at doses between 1 and 150 ng/mL [26]. Therefore, we used low and high doses of leptin, chemerin (10 and 100 ng/mL), and adiponectin (10 and 500 ng/mL) in order to compare our results with available research reports. The chosen high dose of adiponectin (500 ng/mL) differed from the high concentrations of the other two investigated adipokines, to more closely reflect the concentration of adiponectin that can be detected in bovine milk (600 ± 30 ng/mL) [26].

In the next step, we analyzed the expression of genes encoding specific adipokine receptors in BME-UV1 cells. These bovine mammary epithelial cells expressed both types of adiponectin receptors (AdipoR1 and AdipoR2), which agrees with other published studies [16,37]. The results of other research groups indicate that adiponectin may control the expression of its specific receptors by means of a negative feedback regulation; however, it was not observed in our study. Previously, Ohtani et al. [16] investigated the expression of adiponectin and adiponectin receptors in bovine mammary glands at different stages of lactation. The group detected the expression of adiponectin mRNA in the mammary gland, that was significantly lower during lactation. The expression of *ADIPOR1* mRNA showed an opposite tendency to adiponectin, with increased levels at lactation compared to the expression detected in the mammary glands of non-pregnant cows or animals at the dry-off period. *ADIPOR2* expression was also the highest in the mammary gland tissue collected at the late stage of lactation [16]. These results cannot be directly compared with the results of our study. In the case of BME-UV1 cells, the expression of both receptors, *ADIPOR1* and *ADIPOR2*, was more stable, and was not significantly affected by the presence of adiponectin in the culture medium.

Despite the detection of adiponectin receptors in BME-UV1 cells, we did not observe a pronounced effect of adiponectin on functional differentiation or apoptosis induction in bovine MECs. Adiponectin did not change the concentration of αS1-casein synthesized by the BME-UV1 cells and secreted to the culture medium. In addition, we did not observe any effect of adiponectin when cells were grown on Matrigel, forming 3D spherical structures. Previously, Jeong and coworkers [26] reported increased proliferation and cell cycle progression in MAC-T cells treated with adiponectin (20 ng/mL). Interestingly, the stimulatory effect of this adipokine on the proliferative activity of MAC-T cells was diminished at higher doses (100–150 ng/mL). The research group also demonstrated that adiponectin (20 ng/mL) reduced the effect of tunicamycin-induced endoplasmic reticulum stress in MAC-T cells [26]. These results indicated that adiponectin may induce signaling pathways that stimulate bovine MEC proliferation, thus increasing MEC numbers during bovine mammary gland development. However, the most effective dose of adiponectin (20 ng/mL) used in this study was quite low, considering that the concentration of adiponectin in circulating blood in cattle ranges between 10 µg/mL in calves after birth and around 30 µg/mL during the estrous cycle in heifers [28,38]. Our study did not detected any significant changes in the viability of BME-UV1 cells in the presence of low doses (10–50 ng/mL) and higher doses (100–500 ng/mL) of adiponectin. The adipokine did not induce a proapoptotic effect when used at low and high concentrations (10, 500 ng/mL). Different observations were published by Esper et al. [17], who performed an in vitro study on normal human breast epithelial stem cells and showed that adiponectin (used at a concentration of 25 µg/mL) promoted the apoptosis or quiescence of primary stem cells, suppressing the breast stem cells’ self-renewal activity. Similar observations were reported using cultures of MCF-10A normal human mammary epithelial cells and HC11 murine mammary epithelial cells, confirming the antiproliferative and proapoptotic effect of adiponectin [39,40]. We did not observe the induction of apoptosis in the presence of 10 or 500 ng/mL of adiponectin, so the proapoptotic effect described by other research groups may be induced at higher concentrations of adiponectin.

The BME-UV1 cell line also expressed two chemerin receptors: CMKLR1 and GPR1. The expression of *GPR1* mRNA was significantly increased in the presence of low and high doses of chemerin, whereas the level of *CMKLR1* transcript was not significantly affected by chemerin, and was lower compared to the *GPR1* expression. Previously, Suzuki et al. [18] reported that the MAC-T cell line expresses the transcripts of *CMKLR1* and *CCRL2*, but the expression of *GPR1* has not been confirmed. Differences in the results obtained in our research and in the study by Suzuki and coworkers [18] may be connected with the characteristics of the cells used. Both cell lines, MAC-T and BME-UV1cells, were established from primary bovine mammary epithelial cells stably transfected with simian virus 40 thermolabile large T antigen [24,41]. These cell lines can synthesize and secrete milk proteins. However, a unique feature of BME-UV1 cells is the responsiveness to epidermal growth factor [24].

The present research demonstrated that chemerin significantly affected the secretory activity of bovine mammary epithelial cells. BME-UV1 cells showed an increased secretion of αS1-casein upon incubation with the high concentration of chemerin (100 ng/mL). The amount of αS1-casein detected in the culture medium collected from BME-UV1 cells 24 h after treatment with chemerin was similar to the concentration detected in cells exposed to prolactin, and differed significantly from untreated control, as well as other experimental conditions. Suzuki et al. [18] previously demonstrated that chemerin (100 ng/mL) induced the expression of the κ-casein gene (*CSNK*) in MAC-T cells. Our results are in agreement with the hypothesis stating that chemerin may be an important regulator of the bovine mammary epithelial cells’ functional differentiation during lactogenesis. Our observations and the results of other research groups suggest that future studies should focus on the relationship between signals induced by chemerin and other bioactive molecules (hormones and growth factors), because chemerin seem to be one of the important regulators of the bovine mammary epithelial cell functions. Determining the type of interactions between chemerin and other hormones and growth factors is necessary to fully understand the complex nature of the mammary gland development and remodeling.

In the case of leptin, our study did not confirm the expression of leptin receptor on the transcript level, but detected leptin receptor protein (OB-R) in BME-UV1 cells using immunofluorescence staining. The primers used in our study did not allow for a distinction between the isoforms of leptin receptor (OB-Ra to OB-Rf), but were designed for detection of a common region for the different isoforms (based on Bos taurus LepR mRNA, accession number: NM_001012285.2; with the commercial primers, the Ensembl no. ENSBTAG00000005910, transcript ID: ENSBTAT00000007764.5). Some contradictory results can be found in the case of in vitro studies on leptin expression and effect in the bovine mammary epithelial cells. Silva and coworkers [42] detected mRNA of the long form of leptin receptor (*OB-Rb*) in MAC-T cells, and showed that leptin decreased DNA synthesis in cells cultured in medium supplemented with IGF-I or 1% FBS. Feuermann et al. [38] used an in vitro culture of bovine mammary tissue explants, and showed that the expression of leptin receptor mRNA was increased 25 times after addition of prolactin (1 µg/mL) to the culture medium. Further studies of this research group confirmed that leptin alone did not directly regulate bovine MEC function, but in the presence of prolactin, this adipokine enhanced the synthesis of fatty acids and milk proteins, increased MEC proliferation, and decreased the level of apoptotic proteins (cleaved caspase-3) [25,27,38]. On the other hand, Thorn and coworkers [43] did not detect the expression of *OB-Rb* transcript in MAC-T cells and did not confirm the attenuation of the IGF-I-mediated proliferation of these mammary epithelial cells by leptin (used in a concentration of 100 ng/mL). In our study, leptin administered at concentrations of 10 or 100 ng/mL did not cause significant changes in viability, the number of apoptotic cells, or the expression of apoptotic markers (bax and cleaved caspase-3) in BME-UV1 cells. Leptin did not affect the concentration of αS1-casein measured in the conditioned media collected after 24 h culture of BME-UV1 cells, or the formation of mammospheres on Matrigel either. We used a simple experimental model in which bovine MECs were treated with a single adipokine, and co-incubation with other hormones or growth factors was not implemented. It is possible that the effect of leptin on the bovine mammary gland is not direct, and needs to be accompanied by signals induced by other hormones and growth factors (e.g., prolactin, growth hormone, and IGF-I) found in the local microenvironment, or mediated by stromal cells surrounding the mammary epithelium.

In conclusions, our research confirmed the expression of transcripts of both adiponectin receptors (*ADIPOR1* and *ADIPOR2*) and the two chemerin receptors (*GPR1* and *CMLKR1*) in BME-UV1 cells. Leptin receptor protein was detected in bovine mammary epithelial cells by means of immunofluorescence staining. Chemerin (100 ng/mL) caused a significant increase in the concentration of αS1-casein secreted by the BME-UV1 cells, supporting the hypothesis about the role of chemerin as a potent regulator of the mammary epithelium functional differentiation. Leptin and adiponectin did not affect the viability and secretory activity of BME-UV1 cells. It is possible that leptin and adiponectin play a role in the regulation of the bovine mammary epithelial cells’ metabolism and function, acting simultaneously with other endocrine and paracrine factors found within the mammary parenchyma. Future studies on the role of adipokines in the mammary gland development should include more complex experimental models of the co-treatment of bovine MECs with other growth factors and hormones, or a co-culture with different stromal cells, to determine the most important types of interactions existing between the mammary epithelium and the surrounding microenvironment.

## 4. Materials and Methods

### 4.1. Media and Reagents

Dulbecco’s Modified Eagle Medium: Nutrient Mixture F-12 (DMEM/F12), heat-inactivated fetal bovine serum (FBS), penicillin-streptomycin, gentamycin, amphotericin B, secondary antibodies conjugated with Alexa Fluor 488 (cat. no: A21441), Alexa Fluor™ 594 Phalloidin (cat, no: A12381), Hoechst 33342 (cat. no: H3570), and Alexa Fluor^®^ 488 annexin V/Dead Cell Apoptosis Kit (cat. no: V13245) were purchased from Thermo Fisher Scientific (Waltham, MA, USA). Phosphate-buffered saline (PBS), dimethyl sulfoxide (DMSO), insulin (cat. no: I6634), hydrocortisone (cat. no: H0396), holo-transferrin (cat. no: T1283), and thiazolyl blue tetrazolium bromide (MTT) were purchased from Sigma-Aldrich (part of Merck KGaA, Darmstadt, Germany). Bovine Casein Alpha S1, ELISA kit (cat. no: MBS9358728) was supplied by MyBioSource, Inc. (San Diego, CA, USA). Chemerin (cat.no: 2325-CM-025), leptin (cat.no: 498-OB-01M), adiponectin (cat.no: 5095-AC-050), and prolactin (cat. no: 682-PL-050) were purchased from R&D systems (Minneapolis, MN, USA).

### 4.2. Cell Culture

The BME-UV1 bovine mammary epithelial cell line was purchased from the Cell Bank of The Lombardy and Emilia Romagna Experimental Zootechnic Institute, Italy. The BME-UV1 cells were cultured in standard growth medium composed of DMEM/F12 supplemented with 10% (*v*/*v*) FBS, insulin (1 μg/mL), hydrocortisone (1 μg/mL), holo-transferrin (5 μg/mL), and antibiotics/antimycotic: penicillin-streptomycin (50 μg/mL), gentamycin (50 IU/mL), and amphotericin B (2.5 μg/mL). Cells were cultured at 37 °C with a 5% CO_2_ atmosphere in a humidified incubator, with the medium replaced every second day. All experiments were performed on cells from passage numbers ≤ 10. To determine the viability of the BME-UV1 cells in the presence of the investigated adipokines, the adipokines were added to the culture medium at a range of concentrations, namely: chemerin: 5, 10, 25, 50, and 100 ng/mL; leptin: 10, 25, 50, 100, and 200 ng/mL; adiponectin: 10, 25, 50, 100, 200, and 500 ng/mL. In later experiments, the BME-UV1 cells were cultured in the medium supplemented with 10 or 100 ng/mL of leptin or chemerin, or with adiponectin at concentrations of 10 or 500 ng/mL. The cells were incubated in the adipokine-supplemented media for 24 h prior further analysis.

### 4.3. Cell Viability Assay

The cell viability of the BME-UV1 cells was determined using the MTT assay. The mammary epithelial cells were seeded onto 96-well plates at a concentration of 2 × 10^4^ cells per well. When cells reached 80–90% confluence, the medium was replaced with the culture medium supplemented with chemerin, leptin, or adiponectin at different concentrations (described in Section 4.2), and the cells were cultured for subsequent 24 h. Cells cultured in standard growth medium were used as control. Next, the cells were incubated with 0.5 mg/mL tetrazolium salt (MTT) diluted in phenol red-free DMEM/F12 medium for 4 h at 37 °C. After a 4 h incubation, the cells were washed with PBS and incubated for 10 min in 100 μL of DMSO to solubilize the formazan crystals. The absorbance of solubilized formazan crystals in each sample was measured at 570 nm in a multi-well plate reader (Infinite 200 PRO TecanTM, TECAN, Männedorf, Switzerland). All samples were examined in triplicate, and each experiment was conducted three times. Cell viability was calculated with reference to the values of absorbance measured in control samples. The mean absorbance of control samples was designated as 100% of cell viability. Next, the viability of cells in each experimental condition was calculated using a formula: (Absorbance of sample/Mean Absorbance of control samples) × 100%.

### 4.4. RNA Isolation

After a 24 h incubation in experimental conditions or in the control medium, the medium was removed and the BME-UV1 cells were washed in PBS, suspended in RLT Buffer from the RNeasy Mini Kit (QIAGEN, Venlo, The Netherlands), and stored at −80 °C until further use. Total RNA was extracted from the cells with the RNeasy Mini Kit (cat. no: 74104) purchased from QIAGEN (Venlo, The Netherlands), according to the protocol provided by the producer. RNA concentration and purity were determined spectrophotometrically (Nano-Drop 2000 Spectrophotometer, Thermo Fisher Scientific), and the quality was confirmed using microcapillary electrophoresis (Bioanalyzer 2100, Agilent Technologies, Santa Clara, CA, USA).

### 4.5. RNA Reverse Transcription and Real-Time Quantitative PCR

A constant amount of 2 μg of isolated total RNA was reverse-transcribed to cDNA with a High Capacity cDNA Reverse Transcription Kit (Applied Biosystems, Thermo Fisher Scientific, Foster City, CA, USA), according to the instructions provided by the producer, and the reaction was carried out in a Mastercycler Pro (Eppendorf, Hamburg, Germany).

Real-time PCR was performed using SYBR Select Master Mix (Applied Biosystems, Thermo Fisher Scientific), following the manufacturer’s protocol. Commercial primers (PrimePCRTMSYBR^®^ Green Assays) for the genes *OB-R, ADIPOR1, ADIPOR2, CMLKR1, CCRL2, GPR1*, and *RPS9* were supplied by Bio-Rad (Hercules, CA, USA). In addition, other primers detecting leptin receptor gene and an additional housekeeping gene (*histone*) were chosen based on the available literature [28,29,30], and synthesized by Oligo.pl (Laboratory of DNA Sequencing and Oligonucleotide Synthesis, Institute of Biochemistry and Biophysics (IBB), Polish Academy of Science, Warsaw, Poland). From the two tested housekeeping genes (*histone*, *RPS9*), *histone* was chosen as the most stable gene based on two different algorithms: Genorm and NormFinder. Detailed information about all commercial primers used in this study and primers chosen based on the available literature are listed in Table 1. The real-time PCR reaction was performed using AriaMx Real-Time PCR System (Agilent Technologies, Santa Clara, CA, USA). Cycling conditions started with two initial steps at 50 °C for 2 min and 95 °C for 2 min, followed by 40 cycles composed of a denaturation (95 °C for 15 s), annealing (15 s at a temperature dependent on the pair of primers designed), and extension phase (72 °C for 1 min). Relative gene expression was calculated using the 2^−ΔΔCt^ method [44], in which the analyzed genes were normalized to the reference histone gene. The experiment was performed 3 times in duplicate.

### 4.6. Annexin V Assay Analyzing the Number of Apoptotic Cells 

The BME-UV1 cells were seeded onto 6-well plates at a concentration of 1 × 10^5^ cells per well, and were grown until 70% confluence. Next, the medium was replaced with culture medium supplemented with chemerin, leptin, or adiponectin added at the concentrations indicated in Section 4.2, and the cells were cultured for a subsequent 24 h. Cells cultured in standard growth medium were used as control. The cells were trypsinized and centrifuged at 4 °C for 3 min at 1000× *g*. Pellets were washed in 1 mL of ice cold PBS and centrifuged again at 4 °C for 3 min at 1000× *g*. Next, the cells were resuspended in 100 µL of 1X annexin-binding buffer containing 5 μL Alexa Fluor^®^ 488 annexin V (Component A) and 1 µg/mL propidium iodide (PI), and incubated for 15 min, following the producer’s instructions (Alexa Fluor^®^ 488 annexin V/Dead Cell Apoptosis Kit, Molecular Probes, Thermo Fisher Scientific, Waltham, MA, USA). The reaction was terminated by adding 400 μL of 1X annexin-binding buffer, and the samples were mixed gently and analyzed using the FACSAria II flow cytometer (BD Biosciences, Franklin Lakes, NJ, USA). Cells showing a positive staining for Annexin V and negative for PI represented early apoptotic cells; cells showing double positive staining for Annexin V and PI represented late apoptotic cells; and necrotic cells were represented by cells negative for Annexin V and positive for PI. The number of apoptotic cells (Annexin V^pos^/PI^neg^ and Annexin V^pos^/PI^pos^) was presented as a percentage of the total cell count. At least 2 × 10^4^ events were recorded per sample. The data were collected from three independent experiments.

### 4.7. Western Blot Analysis

Protein extraction was performed by lysing the cells with RIPA buffer (cat. no. R0278, Sigma-Aldrich, Merck, Darmstadt, Germany), supplemented with protease inhibitor cocktail (cat. no. P8340) and phosphatase inhibitor cocktail (cat. no. P5726; Sigma-Aldrich, Merck). Cell lysis was carried out for 30 min on ice. Next, the samples were centrifuged for 20 min at (20,000× *g*), and supernatants were collected. Protein concentration in the lysates was determined using Bio-Rad Protein Assay Dye Reagent, according to the producer’s instructions (Bio-Rad Laboratories Inc., Hercules, CA, USA). Proteins (20 μg) were resolved using SDS-PAGE and transferred onto a low-fluorescence PVDF membrane (Merck/Sigma-Aldrich). For immunostaining, the membranes were blocked with 5% nonfat dry milk in TBS (20 mM Tris-HCL, 500 mM NaCl) containing 0.5% Tween20 (TBST buffer). The membranes were incubated at 4 °C overnight, with primary antibodies (anti-cleaved caspase-3 or anti-bax) diluted in blocking buffer. Gapdh was chosen as a reference protein, based on our previous studies with the use of the BME-UV1 cell line [45,46]. Detailed information about the antibodies used in this study is presented in Table 2. On the next day, the membranes were washed three times in TBST buffer and incubated with appropriate secondary antibodies conjugated with IR fluorophores: IRDye^®^ 680 or IRDye^®^ 800 CW, diluted with TBST buffer. The ChemiDoc™ MP Imaging System (Bio-Rad Laboratories) was used to analyze the protein expression. Densitometric analysis was performed using Image Lab 6.1 software (Bio-Rad). Immunoblot analysis was performed in four replicates.

### 4.8. Immunofluorescence Staining and Confocal Microscopy

For the purpose of immunofluorescence staining, the BME-UV1 cells were cultured on chamber slides (Nunc Lab-Tek Chamber Slide System, Thermo Fisher Scientific) in monolayer or 3D cultures on Matrigel. In the case of monolayer culture, cells were grown until 80% confluence and fixed with 3.7% paraformaldehyde (Sigma-Aldrich, Merck) for 15 min at room temperature (RT). In the case of 3D cultures, cells were grown for 11 days on chamber slides covered with Matrigel, following fixation with paraformaldehyde. Next, the cells were permeabilized with 0.5% Triton X-100 diluted with PBS (Sigma-Aldrich, Merck) for 10 min at RT, washed three times in PBS, blocked with 5% normal goat serum for 1 h, and incubated overnight with primary antibodies against leptin receptor (Bioss Antibodies, Woburn, MA, USA) or β1-integrin (Novus Biologicals, Littleton, CO, USA). Detailed information about the antibodies used in this study is presented in Table 2. After overnight incubation with primary antibodies, the cells were washed three times in PBS and incubated with Alexa Fluor 488-conjugated secondary antibodies for 1 h in darkness at RT. In the case of 3D cultures, the cells were also co-incubated with Alexa Fluor 594 Phalloidin, detecting F-actin (cat, no: A12381) (Invitrogen, Thermo Fisher Scientific). Next, the cells were washed three times in PBS, and nuclei were counterstained with 7-amino actinomycin (7-AAD, 5 μg/mL) for 20 min at RT, or Hoechst 33342 (1 μg/mL) for 10 min at RT in darkness. The cells were visualized using a confocal laser scanning microscope FV-500 system (Olympus Optical Co., Hamburg, Germany). In the case of monolayer culture, at least 12 different fields of view (60× objective) were captured per experimental condition. In the case of 3D cultures, images of 5–10 spheroidal structures from each well were taken, and the analysis was performed in three replicates. The diameters of 3D structures formed by the BME-UV1 cells were measured using ImageJ software (https://ij.imjoy.io accessed on 2 April 2024) (National Institutes of Health and the Laboratory for Optical and Computational Instrumentation, University of Wisconsin). The diameters of at least 14 spheroidal structures were measured per experimental condition.

### 4.9. Three-Dimensional Culture of Bovine Mammary Epithelial Cells

The BME-UV1 cells were plated on 8-well chamber slides coated with growth factor-reduced Matrigel (BD Biosciences, Franklin Lakes, NJ, USA). Chamber slides were prepared by covering the surface of each well with 25 µL of Matrigel and incubating the chamber slides at 37 °C for 30 min. Next, cells resuspended in growth medium with the addition of 2% Matrigel were plated at a concentration of 5 × 10^3^ cell/mL on each chamber slide. After 24 h, the medium was replaced with a differentiation medium, containing DMEM/F-12 supplemented with 2% (*v*/*v*) FBS, 2% (*v*/*v*) growth factor-reduced Matrigel, insulin (1 μg/mL), hydrocortisone (1 μg/mL), holo-transferrin (5 μg/mL), and antibiotics/antimycotic: penicillin-streptomycin (50 μg/mL), gentamycin (50 IU/mL), and amphotericin B (2.5 μg/mL). In experimental conditions, the medium was additionally supplemented with chemerin, leptin, or adiponectin added at the concentrations indicated in Section 4.2. The cells were cultured in the described conditions for 11 days. The medium was replaced every second day. On the last day of culture, the cells were fixed with 3.7% paraformaldehyde (Sigma-Aldrich, Merck) for 15 min at RT, washed with PBS, and subjected to immunofluorescence staining, according to the protocol described in Section 4.8.

### 4.10. Immunoenzymatic Assays

The concentration of αS1-casein synthesized and secreted by the BME-UV1 cell line was measured in cells and conditioned media collected after a 24 h culture of MECs in experimental conditions. Prior to sample collection, the cells were incubated with adipokines for 24 h. Cells cultured in standard growth medium were used as the basic control, whereas cells treated with prolactin (PRL, 1 μg/mL) for 24 h were used as a positive control. On the next day, conditioned media were collected and centrifuged at 4 °C for 5 min at 400× *g* to remove the cellular debris. The cells were scraped in cold PBS and centrifuged at 1000× *g*; supernatant was removed and the cells were frozen at −80 °C until further analyses. Protein extraction from the cells was performed using the method described in Section 4.7. Alpha S1-casein concentration in protein extracts and the collected conditioned media was analyzed with the use of immunoenzymatic test Bovine Casein Alpha S1, ELISA kit (MyBioSource Inc., San Diego, CA, USA), according to the protocol provided by the producer. All samples were examined in triplicate and each experiment was conducted three times.

### 4.11. Statistical Analyses

Statistical analyses were performed using GraphPad PrismTM version 7.00 software (GraphPad Software, Inc., La Jolla, CA, USA). One-way analysis of variance (ANOVA) with Dunnett’s multiple comparison post-test was used to determine the significance of effects between the control and experimental treatments. One-way analysis of variance (ANOVA) with Tukey’s multiple comparison post-test was used when analyzing the data from the experiment, determining the concentration of αS1-casein in cells and conditioned media collected after 24 h culture. The effect of adiponectin, chemerin, and leptin was compared to basic control conditions and to the PRL treatment that was used as a positive control (lactogenic hormone inducing milk protein synthesis in MECs). A *p* value of ≤0.05 was considered statistically significant, and *p* ≤ 0.01 or *p* ≤ 0.001 as highly significant.

## Figures and Tables

**Table 1:** Information about the primers used in real-time qPCR analyses.

**Name of Specific PrimePCR™ SYBR^®^ Green Assay**	**Unique Assay ID**	**Annealing Temperature**
OB-R, Cow	qBtaCED0017739	58 °C
ADIPOR1, Cow	qBtaCID0012798	58 °C
ADIPOR2, Cow	qBtaCED0009922	58 °C
CMLKR1, Cow	qBtaCED0011624	58 °C
CCRL2, Cow	qBtaCED0011140	58 °C
GPR1, Cow	qBtaCED0011594	58 °C
RPS9, Cow	qBtaCID0013542	58 °C
**Name of Target gene**	**Nucleotide sequence of primers**	**Annealing Temperature**
BovObR—primer pair 1 ^a^	FRD: 5′-GGACGTTATGAGGCAGTTGT-3′	62 °C
	REV: 5′-GTATGTTCCAGTTTGCACCT-3′	
BovObR—primer pair 2 ^b^	FRD: 5′-GAATGTCATGTGCCTGTGCC-3′	61 °C
	REV: 5′-GGGCTGGACCACGAAATCTT-3′	
BovObR—primer pair 3 ^c^	FRD: 5′-CAATCACTTGCAGGAAGCAA-3′	60 °C
	REV: 5′-TGACACAAGCTGGTGGAGAG-3′	
BovHistone ^a^	FRD: 5′-ACTGCTACAAAAGCCGCTC-3′	60 °C
	REV: 5′-ACTGCCTCCTGCAAAGCAC-3′	

^a^ reference: Sarkar M. et al. (2010) [28]; ^b^ reference: Raza SHA. et al. (2020) [29]; ^c^ reference: Mercati F. et al. (2019) [30].

**Table 2:** List of antibodies used in the study (IF—immunofluorescence staining; WB—Western blot analysis).

**Name of Primary Antibody**	**Producer**	**Catalog Number**	**Dilution**	**Application**
Leptin receptor Rabbit Polyclonal Antibody	Bioss Antibodies	bs-0961R	1:200	IF
Integrin beta 1/CD29 Rabbit Polyclonal Antibody	Novus Biologicals	NBP2-16974	1:200	IF
Cleaved Caspase-3 (Asp175) Rabbit Polyclonal Antibody	Cell Signaling Technology	9661	1:500	WB
Bax (2D2) Mouse Monoclonal Antibody	Cell Signaling Technology	89477	1:500	WB
GAPDH Mouse Monoclonal Antibody	Thermo Fisher Scientific	MA5-15738	1:2000	WB
**Name of Secondary Antibody**	**Producer**	**Catalog Number**	**Dilution**	**Application**
Chicken anti-Rabbit IgG, Alexa Fluor™ 488	Thermo Fisher Scientific	A-21441	1:500	IF
IRDye^®^ 800CW Donkey anti-Rabbit IgG (H + L)	LI-COR	926-32213	1:5000	WB
IRDye^®^ 680LT Donkey anti-Mouse IgG (H + L)	LI-COR	926-68022	1:5000	WB

## Data Availability

The data presented in this study are available on request from the corresponding author. The data are not publicly available due to the continuity of the research on this topic.

## Supplementary Materials

The following supporting information can be downloaded at: https://www.mdpi.com/article/10.3390/ijms25084147/s1.
