# Supplementary material for: Chemerin Stimulates the Secretory Activity of BME-UV1 Bovine Mammary Epithelial Cells
Source: Int J Mol Sci. 2024 Apr 9;25(8):4147. doi: 10.3390/ijms25084147 (PMC11050135; doi:10.3390/ijms25084147)
Supplement: Supplementary file 1 [file ijms-25-04147-s001.zip › ijms-2912462-supplementary.pdf]

Table S1. Data from the experiment determining the concentration of αS1-casein in cells and conditioned media collected after 24h culture was analyzed by one-way analysis of variance (ANOVA) with Tukey's multiple comparison post-test. The effect of adiponectin, chemerin and leptin was compared to basic control conditions and to prolactin (PRL) treatment that was used as a positive control. Table S1 presents information about the adjusted P values for comparisons that were declared statistically significant ($p \leq 0.05$).

**Adjusted P values for experiment determining the concentration of αS1-casein in cells**		
**Tukey's multiple comparisons test**	**Mean Difference**	**Adjusted P Value**
Ctrl. vs. L100	174.7	0.0485
PRL vs. Ctrl.	277.4	0.0426
PRL vs. L10	373.9	0.0014
PRL vs. L100	452.1	<0.0001
PRL vs. A10	362.4	0.0022
PRL vs. A500	291.8	0.0169
Ch100 vs. Ctrl.	273.6	0.0479
Ch100 vs. L10	370.1	0.0016
Ch100 vs. L100	448.3	<0.0001
Ch100 vs. A10	358.6	0.0026
Ch100 vs. A500	288.0	0.0194
A500 vs. L100	160.3	0.0495

**Adjusted P values for experiment determining the concentration of αS1-casein in conditioned media**		
**Tukey's multiple comparisons test**	**Mean Difference**	**Adjusted P Value**
PRL vs. Ctrl.	21.17	0.0181
PRL vs. L10	22.22	0.0052
PRL vs. L100	22.22	0.0052
PRL vs. A10	23.53	0.0025
PRL vs. A500	15.30	0.0159
Ch100 vs. Ctrl.	19.14	0.0251
Ch100 vs. L10	20.19	0.0064
Ch100 vs. L100	20.19	0.0064
Ch100 vs. A10	21.50	0.0030
Ch100 vs. A500	13.27	0.0203
